# Integrating project-based learning-flipped classroom to enhance students' engagement and historical critical thinking in secondary vocational schools

**DOI:** 10.3389/fpsyg.2026.1872661

**Published:** 2026-07-03

**Authors:** Duansheng Huang, Premsuree Chaumthong, Yaoping Liu, Noppavan Namtubtim

**Affiliations:** 1 Department of Education and Society, Institute of Science Innovation and Culture, Rajamangala University of Technology Krungthep, Bangkok, Thailand; 2 Yongkang Hardware Technician College, Yongkang, Zhejiang, China

**Keywords:** flipped classroom, flipped project-based learning, historical critical thinking, project-based learning, student engagement, vocational education

## Abstract

**Introduction:**

This study examined whether the integration of Project-Based Learning and Flipped Classroom into a Flipped Project-Based Learning (FPBL) model was associated with stronger student engagement and historical critical thinking in secondary vocational history learning.

**Methods:**

A quantitative quasi-experimental design with a non-equivalent control group was employed. The participants were 60 eleventh-grade vocational students divided into an experimental group receiving FPBL-based instruction and a control group receiving conventional history instruction. Data were collected across three measurement points using the Student Engagement Scale and the Historical Critical Thinking Skills Inventory.

**Results:**

The experimental group showed stronger improvement than the control group across behavioral, emotional, and cognitive engagement. Similar patterns were found for historical critical thinking, including analyzing historical sources, interpreting and contextualizing historical information, and synthesizing historical arguments. The mixed-design repeated measures ANOVA indicated significant effects of time, group, and time × group interaction.

**Discussion:**

These findings suggest that FPBL may support vocational students' engagement and historical reasoning by connecting digital pre-class preparation, collaborative project based inquiry, and post-class reflection. Because the study used a quasi-experimental design without full random assignment, the findings should be interpreted as evidence of association rather than definitive causality.

## Introduction

The rapid evolution of educational practices in the 21st century demands innovative pedagogical models that foster higher-order thinking, engagement, and learner autonomy (Abas, 2015; Taft, 2015; Nir et al., 2016; Meng et al., 2020). Among these innovations, Project-Based Learning (PBL) and the Flipped Classroom (FC) model have gained prominence for their ability to shift the instructional focus from teacher-centered delivery to student-centered exploration (Tomory, 2022). Project-Based Learning can be defined as a pedagogical approach that engages learners in extended inquiry processes structured around complex, authentic questions and carefully designed products or tasks (Kokotsaki et al., 2016). It emphasizes critical thinking, collaboration, and the application of knowledge to real-world contexts, aligning with constructivist and experiential learning theories. Meanwhile, the Flipped Classroom refers to a blended learning approach in which the traditional order of instruction is reversed: students first engage with instructional materials (e.g., videos, readings) outside class and then apply their understanding through interactive, higher-order learning activities in the classroom (Akçayir and Akçayir, 2018). Integrating PBL with the Flipped Classroom model creates a synergistic learning environment where students actively construct knowledge both inside and outside the classroom.

The integration of these two pedagogical models, referred to as Flipped Project-Based Learning (FPBL)—offers significant benefits for student engagement and historical critical thinking, particularly in secondary vocational schools. Student engagement, defined as the cognitive, emotional, and behavioral involvement in learning activities (Fredricks et al., 2004), is a key predictor of academic achievement and persistence. By combining project-based inquiry with flipped delivery, students are encouraged to take ownership of their learning, collaborate effectively, and connect classroom content with real-life experiences. In history education, where memorization often overshadows analysis, FPBL provides opportunities for students to critically interpret historical sources, evaluate evidence, and understand causality—core elements of historical critical thinking. Historical critical thinking involves interpreting and questioning sources, identifying bias, contextualizing events, and constructing reasoned historical arguments (Burgos-Videla et al., 2025). For vocational learners, who often value practical relevance, these skills foster transferable competencies in reasoning, problem-solving, and civic literacy.

The area of research lies at the intersection of innovative pedagogy, digital learning integration, and historical education in vocational contexts. Recent research conducted by Arifani et al. (2026), as well as Jiang et al. (2026) and Yuan et al. (2026), suggests that educational reform through technological innovation involves more than just the process of delivering the material in terms of pedagogy, as the process of interaction, engagement, and knowledge production is also redesigned. It is important for FPBL in this context, since its contribution lies beyond mere digitization of material presentation. While extensive studies have examined PBL and flipped learning separately (Kokotsaki et al., 2016; Holmes and Hwang, 2016; Rotellar and Cain, 2016; Akçayir and Akçayir, 2018), research on their integration in history subjects—especially within vocational schools—remains limited. Existing research largely focuses on general academic settings (Chua and Islam, 2021), neglecting the unique characteristics of vocational learners who require more applied, contextualized, and collaborative learning experiences (Köpeczi-Bócz, 2024). Moreover, most previous studies have emphasized cognitive outcomes such as knowledge retention or academic performance, while the affective and metacognitive dimensions, particularly engagement and critical thinking—have been less explored.

Despite the extensive research conducted into Project-Based Learning (PBL) and Flipped Classroom (FC), the extant literature does not sufficiently outline how their integration can work as a cohesive approach to instructional methodology in vocational history education. While past studies related to PBL have mainly focused on authentic problem solving, collaboration, and product creation, research related to the Flipped Classroom approach has generally focused on pre-class preparation, online content delivery, and classroom activities. These two lines of studies, however, have not been strongly correlated within history education, especially concerning the secondary vocational schools in which history appears to be a non-technical subject matter with less relevance. The importance of such correlation stems from the nature of history learning, which involves more than mere memorization, including source analysis, context understanding, and argumentative skills. In addition, existing research on PBL and the Flipped Classroom has mainly concentrated on either cognitive performance, motivation, or generic critical thinking, and neglected investigating FPBL in relation to behavioral, emotional, and cognitive engagement and various aspects of critical thinking involved in historical reasoning. As a result, the originality of the current study will stem from its conceptualization of FPBL as an approach involving the integration of pre-class preparation, class project-based history investigation, and post-class reflection for engagement and critical thinking purposes in vocational history education. From a practical perspective, the study will make an empirical contribution in terms of the comparison of FPBL and traditional teaching methods in vocational history classrooms at three different time points.

*Research questions*:

1. How does the integration of Project-Based Learning and Flipped Classroom approaches impact students' behavioral, emotional, and cognitive engagement in history learning at secondary vocational schools?
2. To what extent does the implementation of Project-Based Learning–Flipped Classroom integration enhance students' ability to analyze, interpret, and synthesize historical information critically?

## Literature review

### Theoretical foundation: constructivism, inquiry, and active learning

Both Project-Based Learning (PBL) and Flipped Classroom (FC) are rooted in constructivism theory, which positions students as active subjects in constructing knowledge, not simply recipients of information. Thompson (2010) emphasizes the importance of learning by doing as the foundation for developing meaningful understanding, while Vygotsky and Cole (2018) emphasize the social dimension of learning through collaboration. In the context of FC, the learning process is reconfigured based on Bloom's taxonomy, where lower-level activities (memorizing and understanding) are shifted outside the classroom through videos or online modules, while face-to-face time is used for higher-level activities (analyzing, evaluating, and creating). Meanwhile, PBL focuses on authentic problem-solving through project work that requires in-depth investigation, collaboration, and reflection. The integration of these two approaches creates a learning environment aligned with the principles of active inquiry: students explore real-world problems, examine information from various sources, develop hypotheses, and formulate solutions through social interaction and critical reflection. This orientation is consistent with Dewey's view of reflective learning, in which meaningful understanding develops through inquiry, experience, and the active reconstruction of knowledge (Dewey, 1998).

### Project-based learning: engagement and mastery of 21st-century skills

PBL has been proven effective in increasing learning engagement, creativity, and social responsibility through contextual and meaningful project-based activities. Chimwayange (2025) developed the PBL–Service-Based Skills Development (PBL–SBSD) model, a six-stage cycle (goal setting, research, planning, manufacturing, testing, and modification) that combines real-life problem-solving with community contribution. Through this model, students demonstrate increased behavioral engagement (active participation and persistence), emotional engagement (sense of belonging, pride, and intrinsic motivation), and cognitive engagement (analysis, synthesis, and evaluation). In addition to developing technical skills, PBL–SBSD fosters self-confidence, social empathy, and civic awareness, which are highly relevant in vocational education. Other research (Kokotsaki et al., 2016; Suryanti et al., 2022; Meng et al., 2023) confirms that PBL and problem-solving activities, implemented with effective guidance, can enhance critical thinking, collaboration, and self-efficacy, especially when projects are linked to real-life contexts.

### Flipped classroom: cognitive presence and critical thinking development

Flipped Classroom represents a form of blended learning that places initial understanding outside the classroom and higher-level activities inside. However, various studies have shown that the pre-class phase is often weak in interaction and feedback. Ay and Daghan (2023) addressed this weakness by integrating the Community of Inquiry (CoI) model, consisting of teaching presence, social presence, and cognitive presence, into a Flipped Classroom design. Through a 15-week quasi-experimental study, they found significant improvements in critical thinking strategies and all three dimensions of presence, with critical thinking strategies explaining 60% of the variance in perceived learning community. The Flipped Classroom design, based on the CoI, allows for continuous feedback, collaborative reflection, and social engagement, strengthening metacognitive engagement. The thought-encouraging questions technique by Lee et al. (2024), used in the study, has been shown to stimulate in-depth analysis, logical argumentation, and idea synthesis, making Flipped Classroom not simply a digital content delivery tool but a means of developing higher-order thinking skills. However, flipped classroom implementation may face practical limitations, including limited resources, uneven student preparation, and the need for careful design of pre-class and in-class activities (Stover and Houston, 2019). Similarly, Antonio (2022) showed that the Community of Inquiry framework can be used to evaluate and strengthen flipped classroom learning by emphasizing teaching presence, social presence, and cognitive presence.

### PBL–FC integration in vocational history learning: toward a Flipped Project-Based Learning (FPBL) model

The integration of PBL and FC forms Flipped Project-Based Learning (FPBL), which connects digital-based knowledge acquisition with real-life project implementation. In the pre-class phase, students learn concepts through online videos and modules (the conceptual phase). The face-to-face phase is used to apply the concepts through collaborative projects that require analysis, synthesis, and evaluation (the applicative and reflective phases). This approach addresses two key issues: (1) the gap between theory and practice, by continuously connecting digital activities and real-life projects; and (2) the lack of feedback and social engagement, as the Community of Inquiry principle ensures reflective interaction and guidance throughout the process.

In the context of vocational education, FPBL is highly relevant for history subjects, which are often dominated by factual memorization. With this approach, students not only understand historical events but also develop historical critical thinking—the ability to evaluate sources, recognize bias, understand context, and construct evidence-based arguments (Wineburg, 2010; Freedman, 2015). Through locally context-based projects, such as creating digital exhibitions, narratives of regional history, or reconstructions of social events, students are trained to connect the past with contemporary realities. Chimwayange's (2025) study showed that engaging in meaningful projects fosters intrinsic motivation and social empathy, while Ay and Daghan's (2023). CoI-based FC design fosters in-depth reflection and interaction. The combination of these two makes FPBL an effective means of enhancing behavioral, emotional, and cognitive engagement while developing contextual and reflective historical critical thinking.

The combined analysis of the existing literature suggests that there are both complementary and incomplete strengths in using the pedagogical approach either separately. In particular, the application of PBL enables students to explore, collaborate, and produce their ideas. However, it may turn out to be less effective due to the absence of adequate initial background knowledge as well as feedback from instructors. The FC strategy implies that students can make proper preparation before going to classes and thus makes their class sessions more productive. Yet, the implementation of FC may be hindered by the inability to ensure authentic inquiry and product development during class activities. The problem appears to be relevant for history lessons, which should promote the shift of focus from mere memorization to sourcing, contextualizing, corroborating, and constructing an argument. When it comes to history within vocational settings, the above issue becomes even more important since it requires learning to be practical and collaborative as well as connected with reality. For this reason, the gap identified by this research goes beyond the existing few FPBL studies and refers to the lack of empirical data concerning combining flipped digital preparation and PBL into vocational history lessons.

### Flipped Project-Based Learning (FPBL)

Flipped Project-Based Learning (FPBL) is an integration of two proven effective learning models: Project-Based Learning (PBL) and Flipped Classroom (FC). FPBL emphasizes project-based learning that combines the use of technology in the pre-class phase (Flipped) with the collaborative implementation of real-life projects in the classroom (PBL).

PBL and FC: Two Pillars of FPBL

1. Project-Based Learning (PBL): PBL is a student-centered learning approach in which students work in groups to solve real-life problems with concrete outcomes. This approach encourages the development of critical thinking, problem-solving, and collaboration skills in a real-world context. In the context of history learning, PBL challenges students to analyze, evaluate, and interpret historical sources through relevant projects.
2. Flipped Classroom (FC): FC is a learning approach that reverses the traditional sequence. Students learn basic material first through videos or readings outside of class, while class time is used for discussion, problem-solving, and application of concepts in more in-depth and interactive activities. FC supports more independent and reflective learning by optimizing classroom interactions for the development of higher-order skills.

**Figure 1:**
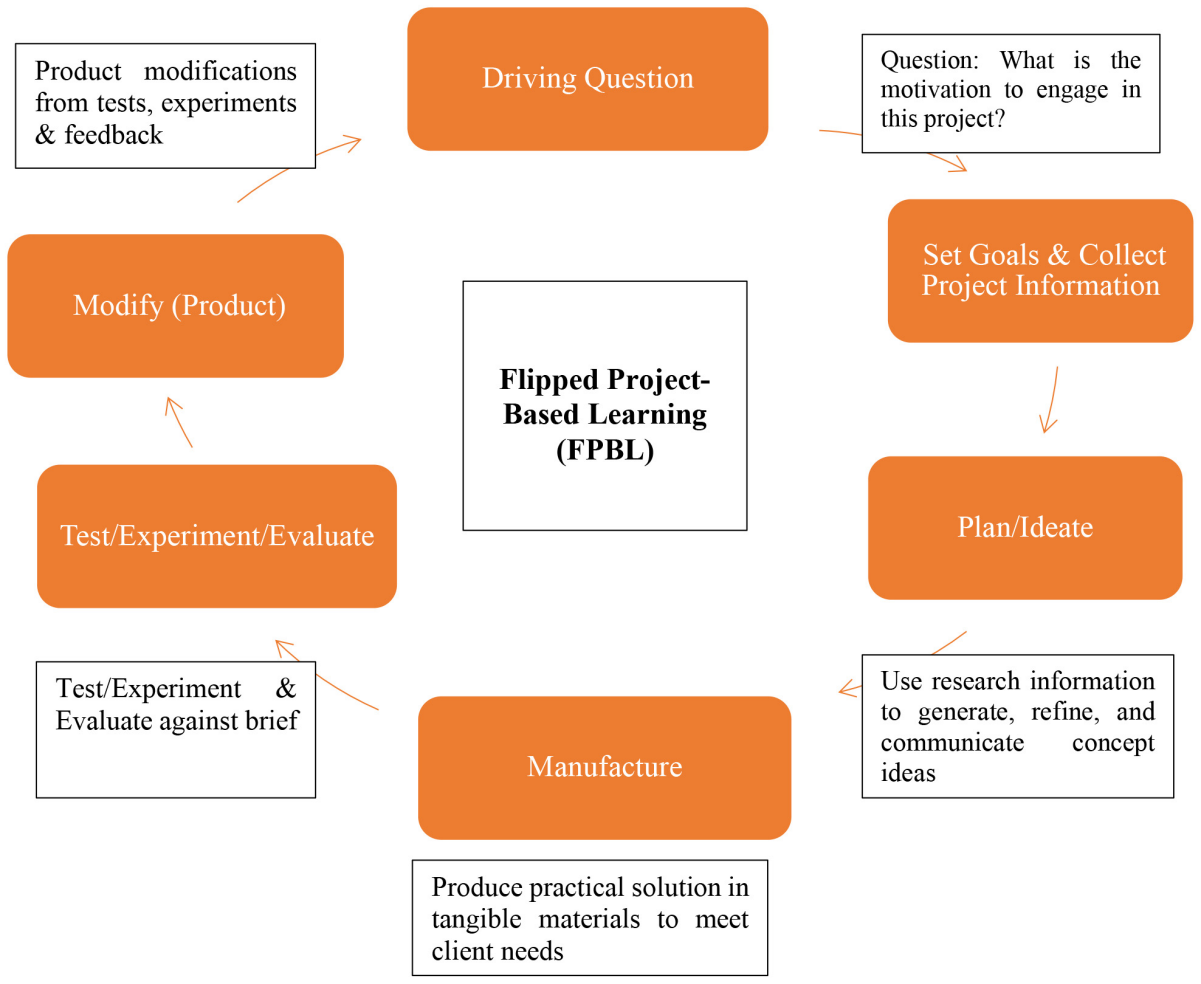
Flipped Project-Based Learning (FPBL) cycle.

The following is a visual model that illustrates the Flipped Project-Based Learning (FPBL) cycle, which combines both approaches in history learning in vocational high schools. The FPBL instructional cycle used in this study is illustrated in Figure 1.

## Methodology

### Research design

The current research utilized a quasi-experimental design using quantitative data to find out whether Flipped Project-Based Learning (FPBL) contributes to improvements in student engagement and historical critical thinking in vocational history courses. Engagement levels among students were measured on behavioral, emotional, and cognitive planes, and students' abilities to analyze historical resources, interpret and place them into context, as well as develop historical arguments, were used to measure their level of historical critical thinking. Quasi-experimental design was implemented because of the nature of classes being used for the experiment, as random assignment of students was not possible. One group of students was taught with the help of FPBL methodology, and another one received regular lessons of history. Both groups were tested before, during, and after the experiment.

### Site selection, sampling technique, and sample

The research location was purposively selected, considering the context of vocational education, where student engagement in learning remains low and pedagogical innovations that integrate theory with practical application are needed. This research was conducted at a public vocational high school with departments in Building and Construction Engineering and Computer and Network Engineering, where history instruction is part of the general curriculum and often perceived as irrelevant by students. Purposive sampling was used to select participants with basic digital literacy skills and minimal experience in collaborative project work from previous learning. A total of 60 students from two eleventh-grade classes were selected as the study sample: 30 students in the experimental group participating in FPBL learning and 30 students in the control group receiving conventional learning. A history teacher experienced in integrating educational technology served as the primary facilitator in the FPBL implementation, while the researcher acted as an observer and mentor during the learning process.

Prior to the study, official permission was obtained from the school principal and the regional education ethics committee, and written informed consent was obtained from the students' parents or guardians. All participants were informed about the purpose of the study, data confidentiality, and their freedom to withdraw at any time during the study. The FPBL implementation process also addressed safety and ethical learning aspects, ensuring that all student history project activities (such as digital artifact creation, mini-exhibitions, and field interviews) complied with school guidelines and did not pose any physical or psychological risks to participants.

### Intervention implementation

The intervention lasted for 8 weeks within normal history classes, having two lessons of 90 min each per week. The study was aimed at creating a clear difference in the manner of instructions given to both experimental and control groups. The experimental group was given instruction using FPBL approach, divided into three stages: pre-class flipped learning, in-class inquiry into projects, and post-class revisions, reflections, and presentations. At the pre-class stage, students were involved in watching brief video tutorials, reading digital resources, analyzing historical sources and answering relevant questions to develop conceptual understanding about the subjects, including historical sources analysis, local and national historical events, concepts of continuity and change, causes and effects of historical events, and significance of historical issues in today's social reality. Then in class, inquiry took place, where students analyzed primary and secondary sources, looked for bias, evaluated evidence, put historical events in context, compared different interpretations, and made evidence-based claims about the history in project works like digital stories, posters, exhibitions, and report writings. At the same time, the teacher facilitated group work and provided formative feedback, as well as encouraging connections between historical knowledge and real life and future vocation. At the post-class stage, students revised their projects according to teacher and peers' feedback, made reflective journals, and presented historical arguments in front of the class, thus reinforcing ownership of the learning process, critical thinking skills, and evidence-based arguments. On the other hand, the control group learned the same topics within the same time frame using the standard approach, given by teachers, textbook reading, question-and-answer exercises, and writing assignments. The weekly structure of the FPBL intervention and the corresponding conventional instruction activities are summarized in Table 1.

**Table 1:** Overview of the FPBL intervention process.

Week	Main topic	FPBL activities in experimental group	Conventional activities in control group
Week 1	Orientation to historical inquiry and project task	Introduction to FPBL, formation of groups, explanation of project goals, introduction to historical source analysis	Teacher explanation of course objectives and textbook-based introduction
Week 2	Identifying historical problems and questions	Students watched pre-class videos, discussed guiding questions, and formulated project research questions	Teacher lecture, textbook reading, and individual question-answer tasks
Week 3	Historical source analysis	Group analysis of primary and secondary sources; identification of bias, reliability, and perspective	Teacher explanation of historical sources followed by individual exercises
Week 4	Contextualizing historical events	Students connected historical events with broader social, cultural, and political contexts	Classroom discussion guided mainly by teacher explanation
Week 5	Constructing historical interpretation	Groups compared evidence, debated interpretations, and drafted historical narratives	Students summarized textbook materials and answered written questions
Week 6	Developing project products	Students developed digital narratives, posters, mini-exhibitions, or group reports	Students completed individual written assignments
Week 7	Revision and feedback	Peer review, teacher feedback, and project revision	Teacher reviewed student answers and provided general feedback
Week 8	Presentation and reflection	Group presentation, reflective journals, and final evaluation	Final class review and individual assessment

### Considerations for internal validity

Considering that the present research employs a quasi-experimental non-equivalent control group design, several steps were implemented to increase internal validity and minimize possible threats. To begin with, the experimental and control groups were selected in such a way as to belong to the same grade level and school in order to minimize contextual differences between the two samples. Secondly, since the same history lessons have been studied by the participants of both groups during the same 8 weeks, the threat posed by history, maturation, and testing was significantly reduced. Thirdly, identical tests have been used when measuring the variables in question during three stages of the experiment. Moreover, since it was impossible to randomly assign particular students to groups due to the intact nature of the latter, they were nevertheless selected in terms of equal academic level, experience of studying the same curriculum, and school learning environment. Finally, treatment integrity was achieved through observing teachers' checklists, their notes, students' learning logs, and project-based products in order to ensure consistent execution of FPBL stages by the former and traditional approach by the latter group of participants. At the same time, results of this group comparison cannot serve as strong evidence that FPBL leads to increased students' engagement and historical reasoning due to the lack of randomization.

### Data collection, analysis and validation

The two quantitative data sets were obtained through administration of two structured instruments, Student Engagement Scale and Historical Critical Thinking Skills Inventory, to the experimental and control groups at the three assessment points: before the start of the intervention, mid-intervention, and end of the intervention. Specifically, the Student Engagement Scale assessed engagement from behavioral, emotional, and cognitive perspectives, while Historical Critical Thinking Skills Inventory evaluated historical critical thinking skills from the perspective of analyzing historical sources, interpreting and contextualizing historical information, and synthesizing historical arguments. The same tools were administered to both groups, with the same procedure and schedule in order to achieve comparability between experimental and control conditions.

The students in the experimental group were oriented to the learning process and project requirements, FPBL structure, digital materials, assigned group roles, and assessment procedures before the actual implementation of the intervention. On the other hand, the control group was oriented only to the history content covered and the assessment procedure. As for the 8-week-long intervention, the experimental group got FPBL instruction while the control group received instruction that was more conventional. The implementation checklists, teaching notes, student learning logs, and project products were employed to ensure fidelity to the treatment assignment, i.e., to ensure that FPBL stages have been implemented as planned and that conventional instruction was delivered consistently throughout the intervention. They will serve no further purpose besides verifying fidelity to intervention.

In order to analyze the quantitative data, descriptive statistics were calculated to show mean scores and standard deviations in student engagement and historical critical thinking between the experimental and control groups at three assessment points. Also, analysis of variance (ANOVA) with mixed design was performed. Specifically, mixed-design repeated-measures ANOVA was carried out to test for time effect, group effect, and interaction between the two variables on students' level of engagement and historical critical thinking. In the model, time was the within-subject variable, while instructional group was a between-subject variable. Pairwise comparisons by Bonferroni correction were employed to detect trends of changes in each group between the three assessments.

### Instrument validity and reliability

Before the data collection phase, the validity and reliability of the instruments were determined. The Student Engagement Scale and the Historical Critical Thinking Skills Inventory were based on existing constructs within student engagement and historical thinking; these instruments were then reviewed by three experts in history education, educational measurement, and vocational pedagogy, who ensured their relevance to the content, clarity, and correspondence to the study aims. Minor changes to the wording, relevance to the context, and correspondence to vocational history learning were made. The pilot test involved students similar to those used in the main study but did not include the latter. Internal consistency was analyzed through Cronbach's alpha. The Student Engagement Scale yielded appropriate results regarding reliability, with Cronbach's alpha equaling 0.82, 0.85, and 0.87 for behavioral engagement, emotional engagement, and cognitive engagement, respectively. Similarly, the Historical Critical Thinking Skills Inventory had appropriate reliability with respect to Cronbach's alpha of 0.84, 0.86, and 0.89, which were found for analyzing historical sources, interpreting and contextualizing historical information, and synthesizing historical arguments, respectively. Thus, it can be argued that both instruments had sufficient validity and reliability.

## Result

Before presenting the results for every research question, it is important to mention that the results have been presented based on an analysis of both the experimental and the control groups. While the experimental group underwent Flipped PBL, the control group was subjected to regular teaching methods. In the earlier draft of the manuscript, the results had been presented in relation to the experimental group only. However, the current draft of the manuscript analyzes the results by comparing the average scores of both groups at the three points of measurement.

*RQ1. The impact of integration of Project-Based Learning and Flipped Classroom approaches toward students' behavioral, emotional, and cognitive engagement in history learning at secondary vocational schools*

The first research question was answered through data obtained from three measurements using the Student Engagement Scale. The average scores for each indicator of student engagement: behavioral, emotional, and cognitive, are presented in Tables 2–4.

**Table 2:** Behavioral engagement item means.

Behavioral engagement items	Group	First measurement	Second measurement	Third measurement
1. I actively participated in class activities.	Experimental	3.62 (1.05)	4.21 (0.88)	4.47 (0.71)
	Control	3.55 (1.02)	3.68 (0.96)	3.76 (0.91)
2. I completed history-related assignments on time.	Experimental	3.58 (0.97)	4.10 (0.82)	4.33 (0.74)
	Control	3.52 (0.95)	3.64 (0.90)	3.72 (0.86)
3. I collaborated effectively with peers during project work.	Experimental	3.47 (1.12)	4.23 (0.91)	4.52 (0.68)
	Control	3.43 (1.08)	3.56 (1.01)	3.63 (0.96)
4. I contributed ideas during group discussions.	Experimental	3.41 (1.09)	4.15 (0.85)	4.44 (0.70)
	Control	3.39 (1.05)	3.51 (0.98)	3.59 (0.94)
Overall mean (behavioral)	Experimental	3.52	4.17	4.44
	Control	3.47	3.60	3.68

According to Table 2, the level of behavioral engagement was higher for the experimental group compared to the control group at all measurement periods. Particularly, the average level in the experimental group was 3.52 at the first period of measurement, and 4.17 and 4.44 at the second and third measurements, respectively. The results show that there was a noticeable increase in the level of students‘ involvement in various activities such as engagement, completion of tasks, cooperation with classmates, and discussion during classwork. In contrast, for the control group, the level of engagement showed relatively low levels, where the average level increased from 3.47 to 3.60 and further to 3.68 at each period of measurement, respectively. It can be concluded that whereas regular teaching helps to promote some levels of behavioral engagement by involving students in routine activities and homework, it is evident that the level of behavioral engagement increased significantly within the FPBL approach. This has been facilitated by the students' preparation before attending class, collaborative work on the historical project, idea negotiation with their peers, and more responsibility regarding project-based learning tasks.

As shown in Table 3, there is clear evidence to suggest that the increase in emotional engagement was higher in the experimental group compared to the control group. In particular, there was an increase in the emotional engagement mean from 3.44 in the first measurement to 4.18 in the second and 4.45 in the third measurement for the experimental group. The trend above clearly shows that there was a higher level of motivation in terms of being emotionally involved in the learning experience, higher levels of positivity toward working on projects together with their peers, as well as feeling proud of what they have learnt. On the other hand, the control group only showed a slight improvement from 3.39 in the first measure to 3.52 in the second measure and to 3.61 in the third measure. It is thus evident that traditional teaching and learning methods can promote a certain level of emotional involvement but lack the passion as compared to the FPBL model. The high level of emotional development among participants in the experimental group can be largely explained by the meaningful steps taken during FPBL, including pre-class preparation, peer collaboration, developing tangible project output, getting feedback, and presentation of work.

**Table 3:** Emotional engagement item means.

Emotional engagement items	Group	First measurement	Second measurement	Third measurement
5. I felt motivated and excited to learn history topics.	Experimental	3.38 (1.14)	4.12 (0.91)	4.36 (0.76)
	Control	3.34 (1.10)	3.49 (1.01)	3.57 (0.96)
6. I enjoyed working on history projects with my peers.	Experimental	3.56 (1.09)	4.19 (0.88)	4.49 (0.72)
	Control	3.50 (1.06)	3.61 (0.99)	3.69 (0.93)
7. I felt connected to the learning activities.	Experimental	3.33 (1.15)	4.08 (0.90)	4.41 (0.79)
	Control	3.30 (1.11)	3.44 (1.03)	3.52 (0.98)
8. I was proud of my project outcomes.	Experimental	3.48 (1.10)	4.31 (0.84)	4.54 (0.69)
	Control	3.42 (1.07)	3.55 (0.98)	3.64 (0.92)
Overall mean (emotional)	Experimental	3.44	4.18	4.45
	Control	3.39	3.52	3.61

As seen in Table 4, there was an observable improvement in cognitive engagement among the experimental group while there were only slight improvements among the control group during the three points of assessment. This was manifested by the rise in the mean score of cognitive engagement of the experimental group from 3.28 in the first point of measurement, which was further improved to 4.11 in the second point of measurement, and finally, up to 4.40 in the third measurement point. In other words, students developed the ability to relate the topics taught in class to the problems in the real world, apply historical concepts into contemporary situations, reflect on their project work, and utilize different sources of information to evaluate historical data. For the control group, however, there was only a small improvement of scores, going from 3.24 in the first point of measurement to 3.39 in the second point and to 3.47 in the third measurement point. This means that even though traditional approach can facilitate knowledge understanding, it is unable to provide sufficient framework for reflection, analysis, and practical application. A mixed design repeated measures ANOVA was run in order to find out how student engagement changed in time during three points of measurements, and also to find out whether any difference could be observed between two groups in terms of changes of student engagement. Specifically, two factors were included into analysis: time (with first, second, and third measurements as levels) and group (two levels: experimental group and control group). The ANOVA was supposed to identify (a) whether the levels of behavioral, emotional, and cognitive engagement had changed over time, (b) whether any differences could be identified between two groups, and (c) whether the pattern of changes had differed depending on type of teaching method (FPBL or conventional instruction). Mauchly's Test of Sphericity indicated that the assumption of sphericity had been violated only for cognitive engagement, χ^2^(2) = 8.41, *p* = 0.01. Hence, the Huynh-Feldt correction was employed. There have been significant effects of time, group, and their interaction on all dimensions of student engagement observed (see Table 5).

**Table 4:** Cognitive engagement item means.

Cognitive engagement items	Group	First measurement	Second measurement	Third measurement
9. I tried to connect historical topics with real-world issues.	Experimental	3.27 (1.13)	4.06 (0.88)	4.35 (0.72)
	Control	3.24 (1.09)	3.39 (1.02)	3.47 (0.96)
10. I applied historical concepts in analyzing current events.	Experimental	3.21 (1.08)	4.10 (0.91)	4.40 (0.73)
	Control	3.18 (1.05)	3.34 (0.99)	3.43 (0.94)
11. I reflected on what I learned after each project task.	Experimental	3.44 (1.02)	4.22 (0.80)	4.47 (0.69)
	Control	3.37 (1.00)	3.50 (0.95)	3.59 (0.90)
12. I used multiple sources to evaluate historical evidence.	Experimental	3.19 (1.17)	4.05 (0.93)	4.38 (0.78)
	Control	3.16 (1.12)	3.31 (1.04)	3.40 (0.98)
Overall mean (cognitive)	Experimental	3.28	4.11	4.40
	Control	3.24	3.39	3.47

**Table 5:** Mixed repeated measures ANOVA results for student engagement.

Engagement dimension	Effect	df	*F*	*p*	η*p*^2^
Behavioral engagement	Time	2, 116	18.64	< 0.001	0.24
	Group	1, 58	12.37	< 0.001	0.18
	Time × Group	2, 116	9.82	< 0.001	0.15
Emotional engagement	Time	2, 116	21.46	< 0.001	0.27
	Group	1, 58	14.08	< 0.001	0.20
	Time × Group	2, 116	10.71	< 0.001	0.16
Cognitive engagement	Time	1.72, 99.76	24.39	< 0.001	0.30
	Group	1, 58	16.25	< 0.001	0.22
	Time × Group	1.72, 99.76	12.84	< 0.001	0.18
Total engagement	Time	1.77, 102.66	26.11	< 0.001	0.31
	Group	1, 58	17.42	< 0.001	0.23
	Time × Group	1.77, 102.66	13.36	< 0.001	0.19

The findings of mixed design repeated measures ANOVA have shown that time played an important role in influencing behavior, emotions, and cognition in terms of engagement. More importantly, the main effect of group as well as interaction between time and group also had a statistically significant effect on behavioral, emotional, and cognitive engagement. Main effect of the group showed that there is a difference in the level of engagement between the experimental and control groups. On the other hand, interaction effect of time and group proved that change in engagement over time was more apparent in the experimental group than the control group. These findings confirm the findings of the descriptive analysis presented in Tables 2–4 showing the improvement of behavioral, emotional, and cognitive engagement in the experimental group compared to the control group. It must be noted that these improvements in engagement levels could not have been achieved simply through repeated lessons in history but rather through the application of FPBL where students undergo digital pre-class preparation and project-based inquiry during class hours.

In order to illustrate the changes occurring in each sample, Bonferroni-corrected pair-wise comparisons were run for the experimental and control groups separately (See Table 6). Based on these analyses, one may conclude that there were statistically significant increments of behavior, emotion, and cognitive engagement from the initial to second measures, as well as from the initial to third measures among students in the experimental group. Nevertheless, increments between the second and third measures did not reach statistical significance; thus, it may be said that the main increment took place at the beginning stage of FPBL implementation and persisted through the last measure. In contrast to the experimental group, in the control group no significant increments could be detected.

**Table 6:** Bonferroni pairwise comparison results for student engagement.

Engagement Type	Group	(*I*) Time	(*J*) Time	Mean difference (*I*–*J*)	Standard error
Behavioral	Experimental	1	2	−0.65^*^	0.18
Behavioral	Experimental	1	3	−0.92^*^	0.17
Behavioral	Experimental	2	3	−0.27	0.15
Behavioral	Control	1	2	−0.13	0.12
Behavioral	Control	1	3	−0.21	0.13
Behavioral	Control	2	3	−0.08	0.11
Emotional	Experimental	1	2	−0.74^*^	0.19
Emotional	Experimental	1	3	−1.01^*^	0.16
Emotional	Experimental	2	3	−0.27	0.14
Emotional	Control	1	2	−0.13	0.13
Emotional	Control	1	3	−0.22	0.14
Emotional	Control	2	3	−0.09	0.12
Cognitive	Experimental	1	2	−0.83^*^	0.20
Cognitive	Experimental	1	3	−1.12^*^	0.18
Cognitive	Experimental	2	3	−0.29	0.16
Cognitive	Control	1	2	−0.15	0.13
Cognitive	Control	1	3	−0.23	0.14
Cognitive	Control	2	3	−0.08	0.12

^*^p<*0.05*.

From the perspective of the Bonferroni-adjusted pairwise comparison results, the experimental group showed significant enhancements for all engagement dimensions assessed at Time 1 in comparison with Time 2 and Time 3. In particular, behavioral engagement was found to be significantly enhanced in relation to Time 2 from Time 1 (MD = −0.65, *p* = 0.001) and Time 3 (MD = −0.92, *p* < 0.001). The same pattern was observed for emotional engagement when comparing Time 1 with Time 2 (MD = −0.74, *p* < 0.001) and Time 3 (MD = −1.01, *p* < 0.001). Lastly, there was a marked increase in cognitive engagement when comparing Time 1 with Time 2 (MD = −0.83, *p* < 0.001) and Time 3 (MD = −1.12, *p* < 0.001). It should be emphasized, however, that the difference between Time 2 and Time 3 was not statistically significant for any dimension, which means that the most considerable improvement occurred during the early/middle stages of the implementation of the FPBL approach. Contrary to this, the control group had insignificant shifts for all engagement dimensions under consideration.

This implies that the experiment group had better results in terms of higher and sustainable engagement, motivation, and reflection compared to the control group. Initially, the level of engagement between the groups was almost equal; yet, after introducing flipped preparation and project-based inquiry, the participants in the FPBL group became highly behaviorally, emotionally, and cognitively engaged. Behavioral engagement grew due to the fact that the students became more independent while working on project tasks, participating in group discussion, working with peers, and conducting historical inquiry projects. Students became more emotionally engaged as they gained an increased sense of ownership, pride, and identification associated with the creation, editing, and presentation of project products. The highest increase in engagement was recorded among cognitive skills, with the participants demonstrating greater competence in making connections between past events and the present, self-reflection, and utilizing different sources to support evidence-based reasoning. The limited change in the level of engagement among the control group could be explained by traditional teaching methods which included mostly teacher-led instruction, textbook reading, answering questions, and completing tasks individually. Thus, in regard to research question 1, it can be concluded that FPBL was superior to conventional instruction in enhancing students' behavioral, emotional, and cognitive engagement. It seems that it happened because FPBL included a holistic process of learning during which students built conceptual readiness based on digital resources and then practiced historical concepts through project-based inquiry.

*RQ2. To what extent does the implementation of Project-Based Learning–Flipped Classroom integration enhance students' ability to analyze, interpret, and synthesize historical information critically?*

Research Question Two addressed the extent to which FPBL contributed to the development of students' historical critical thinking skills compared to traditional teaching approaches. Historical critical thinking skills were examined using the HCTSI, an adaptation of Wineburg's (2010) and Seixas and Morton's (2013) inventory at three different points: before intervention, during intervention, and after intervention. The HCTSI measures the historical critical thinking skills of students along three different scales: analysis of sources, interpretation and context of historical information, and synthesis of historical reasoning. As the study involved a non-equivalent control group quasi-experimental approach, the findings are presented as differences between the experimental and control groups over the three different times of data collection. Mean and standard deviation of each scale is presented in Tables 7–9, while mixed-design repeated measures ANOVA and *post-hoc* analysis (Bonferroni adjustment) are reported in Tables 10, 11.

**Table 7:** Analyzing historical sources item means.

Indicators	Group	First measurement	Second measurement	Third measurement
1. I can identify bias and perspective in primary historical sources.	Experimental	3.15 (1.14)	4.02 (0.90)	4.36 (0.71)
	Control	3.12 (1.10)	3.29 (1.02)	3.38 (0.96)
2. I can evaluate the reliability of different types of historical evidence.	Experimental	3.21 (1.11)	4.08 (0.85)	4.41 (0.72)
	Control	3.18 (1.08)	3.34 (1.00)	3.43 (0.94)
3. I can distinguish between fact, opinion, and interpretation in historical texts.	Experimental	3.09 (1.09)	4.14 (0.88)	4.44 (0.69)
	Control	3.07 (1.06)	3.25 (1.01)	3.35 (0.95)
4. I can use multiple sources to verify historical claims.	Experimental	3.17 (1.13)	4.19 (0.82)	4.47 (0.65)
	Control	3.14 (1.09)	3.31 (0.98)	3.41 (0.92)
Overall Mean (analyzing sources)	Experimental	3.16	4.11	4.42
	Control	3.13	3.30	3.39

**Table 8:** Interpreting and contextualizing historical information.

Indicators	Group	First measurement	Second measurement	Third measurement
5. I can explain events by connecting them with broader historical contexts.	Experimental	3.23 (1.05)	4.15 (0.84)	4.46 (0.67)
	Control	3.20 (1.02)	3.37 (0.97)	3.46 (0.91)
6. I can interpret causes and consequences of major events accurately.	Experimental	3.11 (1.08)	4.13 (0.87)	4.40 (0.70)
	Control	3.09 (1.05)	3.27 (0.99)	3.36 (0.93)
7. I can connect historical themes to social or political developments today.	Experimental	3.18 (1.14)	4.09 (0.90)	4.38 (0.74)
	Control	3.15 (1.10)	3.32 (1.02)	3.40 (0.96)
8. I can recognize continuity and change across historical periods.	Experimental	3.25 (1.10)	4.21 (0.88)	4.49 (0.68)
	Control	3.22 (1.07)	3.41 (0.98)	3.50 (0.92)
Overall mean (interpretation and contextualization)	Experimental	3.19	4.15	4.43
	Control	3.17	3.34	3.43

**Table 9:** Synthesizing historical arguments.

Indicators	Group	First measurement	Second measurement	Third measurement
9. I can synthesize information from multiple historical sources.	Experimental	3.13 (1.12)	4.07 (0.89)	4.40 (0.70)
	Control	3.10 (1.08)	3.28 (1.01)	3.37 (0.95)
10. I can construct logical arguments based on evidence.	Experimental	3.10 (1.09)	4.12 (0.86)	4.44 (0.68)
	Control	3.08 (1.05)	3.26 (0.99)	3.35 (0.94)
11. I can support my conclusions with valid and relevant historical evidence.	Experimental	3.17 (1.15)	4.20 (0.83)	4.48 (0.66)
	Control	3.14 (1.10)	3.33 (0.98)	3.42 (0.92)
12. I can communicate my historical reasoning clearly and coherently.	Experimental	3.09 (1.12)	4.18 (0.84)	4.45 (0.69)
	Control	3.07 (1.08)	3.24 (1.00)	3.34 (0.95)
Overall mean (synthesis)	Experimental	3.12	4.14	4.44
	Control	3.10	3.28	3.37

**Table 10:** Mixed repeated measures ANOVA results for historical critical thinking.

Critical thinking dimension	Effect	df	*F*	*P*	η*p*^2^
Analyzing historical sources	Time	2, 116	25.42	< 0.001	0.31
	Group	1, 58	18.36	< 0.001	0.24
	Time × Group	2, 116	14.27	< 0.001	0.20
Interpretation and contextualization	Time	2, 116	27.18	< 0.001	0.32
	Group	1, 58	19.74	< 0.001	0.25
	Time × Group	2, 116	15.06	< 0.001	0.21
Synthesizing historical arguments	Time	1.74, 100.92	29.63	< 0.001	0.34
	Group	1, 58	21.48	< 0.001	0.27
	Time × Group	1.74, 100.92	16.82	< 0.001	0.23
Total historical critical thinking	Time	1.75, 101.50	31.25	< 0.001	0.35
	Group	1, 58	22.67	< 0.001	0.28
	Time × Group	1.75, 101.50	17.94	< 0.001	0.24

**Table 11:** Bonferroni pairwise comparison results for historical critical thinking.

Critical thinking dimension	Group	(I) Time	(J) Time	Mean difference (I–J)	Standard error	*p*
Analyzing sources	Experimental	1	2	−0.95^*^	0.18	< 0.001
Analyzing sources	Experimental	1	3	−1.26^*^	0.17	< 0.001
Analyzing sources	Experimental	2	3	−0.31	0.15	0.061
Analyzing sources	Control	1	2	−0.17	0.13	0.194
Analyzing sources	Control	1	3	−0.26	0.14	0.073
Analyzing sources	Control	2	3	−0.09	0.12	0.456
Interpretation and contextualization	Experimental	1	2	−0.96^*^	0.17	< 0.001
Interpretation and contextualization	Experimental	1	3	−1.24^*^	0.16	< 0.001
Interpretation and contextualization	Experimental	2	3	−0.28	0.14	0.066
Interpretation and contextualization	Control	1	2	−0.17	0.13	0.201
Interpretation and contextualization	Control	1	3	−0.26	0.14	0.076
Interpretation and contextualization	Control	2	3	−0.09	0.12	0.461
Synthesizing arguments	Experimental	1	2	−1.02^*^	0.18	< 0.001
Synthesizing arguments	Experimental	1	3	−1.32^*^	0.17	< 0.001
Synthesizing arguments	Experimental	2	3	−0.30	0.15	0.063
Synthesizing arguments	Control	1	2	−0.18	0.13	0.176
Synthesizing arguments	Control	1	3	−0.27	0.14	0.069
Synthesizing arguments	Control	2	3	−0.09	0.12	0.454

*p* ^*^ < 0.05.

According to Table 7, the experimental group experienced more significant improvements in historical sources' analysis skills in comparison with the control group. In particular, the mean score of the experimental group increased from 3.16 on the first test to 4.11 on the second one and reached the maximum point of 4.42 on the third assessment. These results mean that the experimental group improved its ability to determine bias and perspectives in primary sources; evaluate the credibility of historical evidence; discern among fact, opinion, and interpretations; and prove historical statements based on multiple sources. In turn, the control group made small progress, raising the mean scores from 3.13 to 3.30 and then from 3.30 to 3.39 on the third test. Therefore, such results indicate that traditional learning may help students understand historical information better; however, it does not provide many opportunities to evaluate sources effectively. The higher progress achieved by the experimental group is possible due to the implementation of FPBL. Within FPBL, students worked with historical sources outside the classroom, evaluated evidence while working on the project, compared perspectives of sources, and supported their historical knowledge through the investigation of evidence.

As seen from Table 8, the experimental group showed better development in terms of interpreting and contextualizing historical data than the control group. Overall scores increased gradually from 3.19 at Assessment 1 to 4.15 at Assessment 2 and to 4.43 at Assessment 3 in the experimental group, which means that students had developed skills in describing historical events in relation to their context, analyzing causes and consequences, making connections between historical topics and the current socio-political situation, and identifying continuity and changes in history. Meanwhile, a slight improvement was seen in the control group, where the overall score increased from 3.17 to 3.34 at Assessment 1 and then to 3.43 at Assessment 3. It could be inferred that traditional teaching allows for an understanding of historical data and chronological relationships; however, students do not have many opportunities to learn how to interpret historical events or analyze them historically. The higher progress of the experimental group shows that using FPBL helped students move beyond factual knowledge toward contextualization as they discussed historical issues, evaluated different perspectives on events, made connections between past and present, and provided explanations for historical projects.

From Table 9 it can be stated that the experimental group showed significantly greater development in the ability to synthesize historical arguments. In particular, for the experimental group, their average scores were 3.12, 4.14, and 4.44, respectively, on measures one, two, and three. These numbers show that students from the experimental group have become better in synthesizing information from various historical texts, making logically supported claims on the basis of facts, supporting claims with valid and relevant information from historical sources, and making clear statements on the basis of reasoning related to the material under discussion. At the same time, the control group only showed minor improvements, from 3.10 to 3.28 and then to 3.37 on the respective measures. Hence, it is possible to assume that the traditional method of teaching helps students summarize historical facts, but it does not assist them much in creating their own arguments. It seems likely that the larger improvement was achieved by the experimental group because FPBL encourages students to synthesize information from different sources, argue their points with others, reconsider their arguments based on feedback, and present project-based historical explanations.

An analysis of variance with mixed design and repeated measures was performed to check the significance difference between the improvement in historical critical thinking between the experimental and control groups. The repeated measures variable, time, involved the first, second, and third measurement periods, while the grouping variable included the experimental and control groups. Three types of effect were considered in the analysis: effect of time, effect of group, and time × group effect. The results from Mauchly's Test of Sphericity showed that the assumption of sphericity was not violated for the analysis of historical sources (χ^2^(2) = 4.28, *p* = 0.13) and interpretation and contextualization (χ^2^(2) = 3.96, *p* = 0.14). However, it was violated for the synthesis dimension (χ^2^(2) = 8.67, *p* = 0.01), and thus the Huynh-Feldt correction was used for the latter. According to Table 10, the interaction between time and group variables was statistically significant in all dimensions analyzed (see Table 10).

All dimensions of the historical critical thinking showed a statistically significant time effect, group effect, and time by group interaction effect in the mixed design ANOVA. The time effect indicates changes over time through three observations, group effect refers to the level of experimental and control groups, and the interaction effect indicates that there were more improvements for the experimental group than for the control group. These results agree with the results of Tables 7–9, indicating that the experimental group improved significantly on source analysis, interpretation, and synthesis, whereas the control group did not show much improvement. For further elucidation on the origin of interaction effects, Bonferroni-adjusted *post hoc* tests were carried out separately for the experimental and control conditions (See Table 11). Findings show that there were significant gains for the experimental condition from measurement one to two and from measurement one to three in all aspects of historical critical thinking. Nonetheless, the differences between measurements two and three were not significant, thereby indicating that gains were made in the initial to mid stages of the FPBL treatment and sustained till the last measurement. The control condition showed no significant gain over the three measurements.

Based on the Bonferroni-corrected *post-hoc* pairwise comparisons, there can be noted that development in terms of the use of historical critical thinking occurs among participants within the experimental group. For example, in terms of analyses of historical sources, it has been revealed that within the experimental group there have been statistically significant differences between Time 1 and Time 2 (MD = −0.95, *p* < 0.001) as well as between Time 1 and Time 3 (MD = −1.26, *p* < 0.001). Similar trends have also been detected regarding interpretation and contextualization, where statistically significant differences have been found between Time 1 and Time 2 (MD = −0.96, *p* < 0.001) as well as between Time 1 and Time 3 (MD = −1.24, *p* < 0.001). Meanwhile, the greatest progress has been detected in terms of synthesis of historical arguments; it has also been noted that within the experimental group there were statistically significant differences between Time 1 and Time 2 (MD = −1.02, *p* < 0.001) as well as between Time 1 and Time 3 (MD = −1.32, *p* < 0.001). No statistically significant differences have been observed between Time 2 and Time 3 in terms of three dimensions, which allows concluding that improvements related to historical critical thinking occurred at the early-middle stage of FPBL intervention and persisted until the final measurements. As for the control group, no statistically significant progress has been observed in terms of the use of historical critical thinking.

In summary, it is evident from the findings in relation to Research Question 2 that FPBL was more effective in improving the historical critical thinking skills of the participants when compared to traditional teaching methods. As opposed to the participants in the control group, those in the experimental group showed a marked improvement on all the three dimensions of critical thinking, which included analyzing historical sources, making sense of historical information, and synthesizing arguments. On the contrary, those in the control group showed very minimal changes on all the three points of testing. It is noteworthy that the greatest level of improvement was observed in the synthesis dimension, and this was made possible by the fact that FPBL promoted synthesis of historical evidence and argumentation.

## Discussion

The results show that students who participated in FPBL showed higher improvement in terms of engagement and historical critical thinking compared to the traditional teaching method. At all three testing points, the behavior, emotion, and cognition of students were more involved with respect to the treatment group while only slight improvement was found from the control group. Similarly, historical critical thinking was shown to improve considerably among students in the FPBL condition in terms of source analysis, interpretation and context creation, and historical argument synthesis. The interactions between time and groups further indicated that the results may not be attributed to the repetitive nature of the history class but rather to the specific learning method that was adopted in FPBL. The findings of this research support Alt's claim that active, inquiry-based, and socially-mediated learning environment is superior to teacher-centered education to encourage engagement and critical thinking (Alt, 2015; Vygotsky and Cole, 2018).

### FPBL as a catalyst for multidimensional engagement

According to the findings obtained regarding Research Question 1, there were some differences between the control and experimental groups' levels of multidimensional engagement. Though all classes studied the same historical topics throughout the course, the latter group displayed more progress concerning all three dimensions. The results suggest that flipped peer-based learning created favorable conditions for engaging behaviors, emotions, and thoughts. In terms of behavioral engagement, this model helped promote participation before lessons, active participation in the inquiry process, managing the project process, negotiating with peers, and producing project output. Thus, students became active participants in the historical content creation process. The definition of engagement as a multidimensional construct given by Fredricks et al. (2004) and Suryanti et al. (2019) implies behavioral, emotional, and cognitive involvement. Emotional engagement was found higher among the experimental group members because project-based activities allowed for developing a sense of pride, belongingness, and responsibility for what they were doing. Contrary to that, the control group showed poor results related to emotional involvement in learning activities because the traditional model used was mostly focused on teacher explanations and textbook readings, question-and-answer activities, and individual assignments. Therefore, the current results prove the hypothesis that participating in meaningful activities increases the motivation, sense of responsibility, and cooperation skills (Chimwayange, 2025; Kokotsaki et al., 2016; Meng et al., 2023).

Regarding cognitive engagement, it should be noted that it developed significantly in the experimental group. As part of flipped peer-based learning, this dimension was promoted thanks to the necessity to discuss contemporary topics related to historical facts, analyze the project task, and work with various sources and use them to evaluate the evidence related to the topics under discussion. The pre-class preparation stage and project component correspond to different phases of the Community of Inquiry (Ay and Daghan, 2023; Günbatar, 2021). Namely, teaching presence involved teachers' support and feedback, social presence resulted from group work, and cognitive presence was promoted through the analysis of the sources, discussions, reflections, and projects.

### FPBL as a framework for historical critical thinking

Concerning Research Question 2, the findings suggest that flipped problem-based learning leads to better improvements in students' historical critical thinking than traditional teaching. In particular, while students who received traditional training showed only limited advancements, those who took part in the experiment advanced in their ability to analyze sources, interpret and contextualize them, and construct arguments. This result is important since it implies that historical critical thinking is not developed merely via receiving information, but through an educational setting that enables learners to evaluate evidence, debate interpretations, contextualize events, and create arguments. Such an approach can be viewed as consistent with Wineburg's (2010) definition of historical thinking that is associated with systematic processes of reading, debating, and analyzing data.

Firstly, the improvement of the quality of source analysis indicates that flipped learning helped the participants develop historical skills. In other words, through preparation before class and activities related to projects, students engaged in source identification, detection of bias and perspective, examination of reliability, distinguishing between facts and interpretations, and corroborating statements on the basis of several sources. Such activities correspond to Wineburg's (2010) and Seixas's (2017) description of essential historical skills that comprise sourcing, contextualization, corroboration, and evidence-based analysis. In addition, the lack of progress in this dimension among the control group participants suggests that traditional learning facilitates basic awareness of facts without systematic analysis of sources.

Secondly, the advancement in interpretation and contextualization implies that FPBL promoted the perception of historical events as complex processes embedded into certain contexts. Namely, instead of considering history as the collection of isolated facts, the students were taught to examine causes and consequences of events, detect changes and continuity, and correlate historical themes with current social phenomena. This aspect is particularly important in vocational education where history tends to be perceived by students as irrelevant to practice. The application of FPBL helps overcome the issue by connecting historical studies to real-world problems, discussions, and project-based production thus proving the argument that vocational learning should provide contextualized and applied experience (Köpeczi-Bócz, 2024).

Finally, the most significant advancement observed in this experiment pertains to the development of students' skills to construct arguments based on historical facts. The result implies that flipped learning with a focus on project-based activities was successful in helping students combine different pieces of information into arguments, justify their positions based on reliable evidence, and apply the principles of historical reasoning consistently. From the perspective of this research, the result is important since it suggests that vocational learning in combination with flipped instruction creates favorable conditions for developing historical skills. Moreover, the result is consistent with previous studies conducted by Kim et al. (2017) and Wu et al. (2017).

## Conclusion

This study concludes that the integration of PBL and FC into the Flipped Project-Based Learning model results in higher levels of effectiveness of the proposed approach compared to traditional teaching methods in terms of raising learners‘ engagement and critical thinking abilities in vocational history education. It has been shown that in contrast to the control group, the students of the experimental group have achieved more progress in terms of behavioral, emotional, and cognitive engagement as well as in terms of their ability to analyze, interpret, contextualize, and synthesize the historical data in all three dimensions of historical critical thinking. These findings demonstrate that FPBL creates an atmosphere of active collaboration and reflection, where the students prepare digitally before class, conduct project work based on this knowledge in class, and reflect after class. In this way, the learners were more engaged during lessons, took initiative in their learning process, associated historical issues with practical applications, and created reasonable arguments about the historical events under discussion. These findings confirm that FPBL functions as a transformative learning model that combines inquiry-based learning (PBL) and technology-based reflective learning (FC) to produce a more meaningful learning experience. In the context of vocational education, this model is a pedagogical solution to address low learning motivation in non-technical subjects such as history, while strengthening students' critical thinking competencies and historical awareness.

## Implications

Theoretically, this research contributes to a more detailed application of the Community of Inquiry approach and constructivism as a general learning approach to vocational history education. Relying on previous studies' conclusions about the beneficial impact of flipped learning on students' cognitive presence, interactions, and critical thinking with adequate support from the instructor (Ay and Daghan, 2023; Günbatar, 2021), this research shows how using a combination of flipped learning with inquiry-based projects gives students a better opportunity to engage in the process of history education via three stages: preparation before class, problem-solving activities during class, and reflection, revisions, and presentations. This model is likely to contribute to a stronger connection between digital learning, social interactions, and historical inquiries and to support the constructivist idea that learners create knowledge through interaction and inquiries (Alt, 2015; Vygotsky and Cole, 2018). Methodologically, the results of this research show that FPBL provides more benefits than traditional methods of history instruction do. Although some teachers can encourage active involvement of their students in discussions and improve students' basic comprehension of historical concepts, traditional teaching does not provide sufficient opportunities for evidence-based learning, solving historical problems collaboratively, and presenting and defending historical ideas. With FPBL, however, it is possible to make history lessons more inquiry-based activities because students have the chance to develop both their engagement and critical thinking skills. Therefore, FPBL can be considered a more complex approach than the simple concatenation of two approaches.

## Data Availability

The anonymized datasets supporting the conclusions of this article will be made available by the corresponding author upon reasonable request, without undue reservation.
